# Factors Contributing to Sexual Dysfunction in Female Schizophrenia Patients During Recovery: A Multifactorial Analysis

**DOI:** 10.62641/aep.v53i2.1867

**Published:** 2025-03-05

**Authors:** Jing Liu, Yina Zhang, Xu Mao

**Affiliations:** ^1^Reproductive Medicine Center, The First Affiliated Hospital of Jinzhou Medical University, 121000 Jinzhou, Liaoning, China; ^2^Psychiatric Rehabilitation Department, Jinzhou Kangning Hospital, 121000 Jinzhou, Liaoning, China; ^3^Department of Nursing, The First Affiliated Hospital of Jinzhou Medical University, 121000 Jinzhou, Liaoning, China

**Keywords:** recovery phase schizophrenia, female sexual dysfunction, multifactorial analysis, personalized intervention strategies

## Abstract

**Background:**

Female patients with schizophrenia may experience sexual dysfunction during the recovery period. Therefore, this study conducted a multifactorial analysis to identify factors impacting sexual dysfunction, aiming to aid in developing effective personalized intervention strategies and improving sexual function recovery in these patients.

**Methods:**

This retrospective study included the clinical data from 261 female schizophrenia patients treated at the First Affiliated Hospital of Jinzhou Medical University, diagnosed between February 2022 and March 2024. Based on the total Female Sexual Function Index (FSFI) scores, the patients were divided into the female sexual dysfunction (FSD) group (n = 69) and the non-female sexual dysfunction (non-FSD) group (n = 192). The clinical data of these patients were evaluated using the FSFI, Hamilton Depression Rating Scale (HAMD), Hamilton Anxiety Rating Scale (HAMA), and Olson Marital Quality Questionnaire. Furthermore, univariate and multivariate logistic regression analyses were used to investigate the factors influencing sexual dysfunction in these patients.

**Results:**

The FSD group exhibited significantly lower scores in sexual desire, orgasm, and sexual satisfaction than those in the non-FSD group (*p* < 0.001). Analysis revealed that menstrual status, reproductive history, and mental health status (anxiety and depression) were significantly associated with sexual dysfunction (*p* < 0.05). Furthermore, marital satisfaction, personality compatibility, spousal communication, conflict resolution methods, and sexual life were significantly linked to sexual dysfunction (*p* < 0.05). Additionally, childbirth (Odds Ratio (OR) = 2.531, 95% Confidence Interval (CI) = 1.025–6.25, *p* = 0.044), marital satisfaction (OR = 0.886, 95% CI = 0.824–0.952, *p* = 0.001), conflict resolution methods (OR = 0.816, 95% CI = 0.743–0.897, *p* < 0.001), sexual life (OR = 0.929, 95% CI = 0.876–0.986, *p* = 0.016), anxiety (OR = 1.459, 95% CI = 1.231–1.729, *p* < 0.001), and depression (OR = 1.116, 95% CI = 1.008–1.236, *p* = 0.035) were found as independent influencing factors for sexual dysfunction in these patients during the recovery phase.

**Conclusion:**

Childbirth, anxiety, depression, marital satisfaction, conflict resolution methods, and sexual life serve as independent influencing factors for sexual dysfunction in female schizophrenia patients during the recovery phase. Management approaches targeting these factors can significantly improve sexual dysfunction in this patient population.

## Introduction

Schizophrenia is a complex mental condition characterized by significant impairments in thought, emotions, and behavior that severely disturb a patient’s daily life and interfere with social functioning [1, 2, 3]. Despite significant advancements in optimizing domestic management strategies and psychological interventions, complete recovery in these patients remains a considerable challenge [4]. Studies have reported significantly higher sexual dysfunction among individuals with schizophrenia compared to the general population [5]. However, despite its potential impact on physical and mental health and social functioning, sexual impairments among schizophrenia patients remain unexplored [6].

Sexual dysfunction primarily manifests as decreased libido, orgasmic dysfunction, and problems with sexual intercourse [7]. These issues lead to physical discomfort and can also affect patients’ self-esteem and gender identity, thus increasing emotional problems like anxiety and depression, potentially hindering their psychological recovery [8]. Moreover, it can disrupt emotional communication and intimacy between spouses, weakening the support system provided by families and social networks [9, 10]. This impairment in social adaptability and overall quality of life can collectively extend the recovery process.

From a psychological perspective, sexual dysfunction is closely associated with emotional disorders like anxiety and depression. Anxiety often causes excessive tension, while depression is typically linked to reduced motivation, both of which can reduce libido and sexual satisfaction, thereby aggravating sexual dysfunction. Marital status, a crucial factor in social support, also plays a vital role in the occurrence and progression of sexual dysfunction [11]. Studies have revealed that marital discord, ineffective communication, or inadequate emotional support can promote resistance to intimate behaviors, further worsening sexual dysfunction [12, 13]. For females, childbirth is a significant contributor to sexual dysfunction, as it can result in physiological changes, such as pelvic floor muscle injury and hormonal fluctuations, along with psychological stress and the challenges of transitioning to a new role. These factors adversely impact the quality of sexual life. In individuals with schizophrenia, the influence of childbirth-related challenges may be further intensified by emotional disturbances and cognitive biases, significantly increasing the risk of sexual dysfunction [14, 15].

It is noteworthy that schizophrenia patients without sexual dysfunction during the recovery period demonstrate significantly better overall recovery outcomes compared to those with sexual dysfunction. This suggests that sexual dysfunction adversely impacts recovery across multiple dimensions, including physical health, psychological well-being, and social functioning. Therefore, it is clinically essential to explore the underlying factors contributing to sexual dysfunction, particularly individual differences in psychological state and marital status. This study aims to identify the crucial factors influencing sexual dysfunction in schizophrenia patients, unveil potential underlying mechanisms, and provide a theoretical basis for improving sexual function and overall quality of life. Ultimately, the findings seek to provide a scientific basis for developing tailored intervention strategies.

## Methods

### Research Subjects

A retrospective analysis was conducted on the clinical data obtained from female schizophrenia patients who were treated at the First Affiliated Hospital of Jinzhou Medical University between February 2022 and March 2024. This study was approved by the Ethics Committee of the First Affiliated Hospital of Jinzhou Medical University (No. 202248) and conducted following the principles of the Declaration of Helsinki. Furthermore, informed consent was obtained from all subjects and their parents or guardians.

The inclusion criteria for this study were as follows: (1) female patients aged 20 years or older; (2) patients who showed significant improvement in schizophrenia symptoms after treatment at the First Affiliated Hospital of Jinzhou Medical University; (3) patients with a stable sexual partner exhibiting normal sexual function; and (4) patients capable of understanding and independently completing the questionnaire.

However, individuals who were either pregnant or breastfeeding, those uncooperative with the investigators, and patients with incomplete clinical data were excluded from the final analysis.

### Female Sexual Function Index (FSFI)

The FSFI questionnaire was employed to evaluate female sexual function [16]. This scale assesses six domains: sexual desire, sexual arousal, vaginal lubrication, orgasm, sexual satisfaction, and pain during intercourse. The total score ranges up to 36, with a score of ≤26.55 indicating sexual dysfunction. A higher total score reflects better sexual function. The Cronbach’s α coefficient for the overall FSFI score is 0.882, while the Cronbach’s α coefficients for the individual domains range from 0.722 to 0.898. Based on the FSFI score [17], study participants were divided into two groups: the female sexual dysfunction (FSD) group (FSFI score ≤26.55) and the non-female sexual dysfunction (non-FSD) group.

### Hamilton Depression Rating Scale (HAMD)

The HAMD was used to evaluate depression associated symptoms in patients, including depression, suicidal ideation, guilt, shallow sleep, and agitation across 24 items [18]. It utilizes a five-point scoring system from 0 (normal) to 4 (severe). A total score exceeding 8 suggeststhe presence of depressive symptoms.

### Hamilton Anxiety Rating Scale (HAMA)

The HAMA was used to assess various symptoms in patients, including anxiety, tension, fear, insomnia, and somatic anxiety across 14 items [19]. The scale employs a five-point rating system ranging from 0 (normal) to 4 (severe symptoms). A total score above 7 indicates the presence of anxiety symptoms.

### Olson Marital Quality Questionnaire

The Olson Marital Quality Questionnaire, developed by Fowers BJ and Olson DH [20] at the University of Minnesota, was used to assess marital satisfaction and identify sources of marital conflict. It includes 124 items, covering 12 factors: idealization, marital satisfaction, personality compatibility, communication between spouses, conflict resolution, economic arrangements, leisure activities, sexual life, children and marriage, relationships with relatives and friends, role equality, and consistency of beliefs. Each item is scored on a five-point scale (1–5). The leading statistical indicators include total and factor scores, with higher scores indicating better marital quality.

### Statistical Analysis

Statistical analysis was performed using SPSS 23.0 software (IBM Corporation, Armonk, NY, USA). The normality of continuous variables was assessed using the Shapiro-Wilk test. Continuous variables following a normal distribution were expressed as mean ± standard deviation ($\bar{x}$ ± s), and comparisons between two groups were conducted using the independent sample *t*-test. Non-normally distributed continuous variables were presented as medians with interquartile ranges (minimum, maximum), with group comparisons performed using the Mann-Whitney U test. Moreover, categorical variables were expressed as frequencies and percentages n (%), with comparisons between groups conducted using the chi-square (χ^2^) test. Additionally, multivariate analysis was performed using a binary logistic regression model to identify factors influencing FSD. A *p*-value of <0.05 was considered statistically significant.

## Results

### Baseline Characteristics of the Study Participants

This study included 261 female schizophrenia patients, with a mean age of 43.29 ± 5.60 years and an average body mass index (BMI) of 22.25 ± 1.37 kg/m^2^. The mean total score of the FSFI was 26.53 ± 2.89. However, the average total score of marital quality was 417.62 ± 21.21. Psychological assessments indicated a mean HAMD score of 14.13 ± 3.58 and a mean HAMA score was 12.12 ± 2.37 (Table 1).

**Table 1.** **Analysis of baseline characteristics of the patients [$\bar{x}$ ± s, n (%)]**.

Items		Patient (n = 261)
Age (years)		43.29 ± 5.60
BMI (kg/m^2^)		22.25 ± 1.37
Menstruation	Normal	198 (75.86%)
	Amenorrhea	32 (12.26%)
	Disorder	31 (11.88%)
Educational level	Secondary school and below	146 (55.94%)
	University and above	115 (44.06%)
Monthly household income	<$822	132 (50.57%)
	≥$822	129 (49.43%)
Marriage	Yes	216 (82.76%)
	No	45 (17.24%)
Childbirth	Yes	184 (70.50%)
	No	77 (29.50%)
Past medical history	Yes	202 (77.39%)
	No	59 (22.61%)
FSFI score	Sexual desire	3.95 ± 1.06
	Orgasm	4.62 ± 1.04
	Pain during intercourse	4.54 ± 1.05
	Sexual arousal	4.61 ± 0.98
	Vaginal lubrication	4.56 ± 1.14
	Sexual satisfaction	4.25 ± 1.13
	Total score	26.53 ± 2.89
The Olson Marital Quality Questionnaire score	Idealization	39.57 ± 8.06
	Marital satisfaction	35.64 ± 5.09
	Compatibility	34.53 ± 6.71
	Spousal communication	33.46 ± 6.15
	Conflict resolution methods	33.97 ± 4.46
	Economic arrangements	34.55 ± 6.26
	Leisure activities	34.82 ± 5.85
	Sexual life	35.77 ± 6.13
	Children’s life	35.59 ± 6.08
	Relationships with friends and family	37.88 ± 5.54
	Role equality	30.59 ± 4.37
	Shared beliefs	31.26 ± 5.32
	Total score	417.62 ± 21.21
Psychological status	HAMD score	14.13 ± 3.58
	HAMA score	12.12 ± 2.37

Note: BMI, body mass index; FSFI, Female Sexual Function Index; HAMD, Hamilton Depression Rating Scale; HAMA, Hamilton Anxiety Rating Scale.

### Comparison of Sexual Function between the FSD and Non-FSD Groups

A comparison revealed that the FSD group exhibited significantly lower scores across sexual desire, orgasm, and sexual satisfaction compared to the non-FSD group (*p* < 0.001). However, no significant differences were observed between the two groups in pain during intercourse, sexual arousal, and vaginal lubrication (*p* > 0.05) (Table 2).

**Table 2.** **Comparison of FSFI scores between the FSD and non-FSD groups [M (Q_𝐌𝐢𝐧_, Q_𝐌𝐚𝐱_), $\bar{x}$ ± s]**.

Items	FSD	non-FSD	*z/t*-value	*p*-value
	(n = 69)	(n = 192)		
Sexual desire	3 (1, 6)	4 (2, 6)	4.12	<0.001
Orgasm	4 (1, 6)	5 (2, 6)	6.79	<0.001
Pain during intercourse	4 (2, 6)	5 (1, 6)	0.83	0.41
Sexual arousal	5 (1, 6)	5 (2, 6)	1.47	0.14
Vaginal lubrication	5 (2, 6)	5 (2, 6)	0.95	0.34
Sexual satisfaction	3 (1, 4)	5 (1, 6)	7.80	<0.001
Total score	24.12 ± 2.61	27.40 ± 2.48	9.63	<0.001

Note: FSD, female sexual dysfunction; non-FSD, non-female sexual dysfunction.

### Comparison of General Characteristics between FSD and Non-FSD Groups

The analysis revealed significant differences in menstrual status and childbirth history between the FSD and non-FSD groups (*p* < 0.05). However, no significant differences were observed between the two groups regarding age, BMI, educational level, marital status, monthly household income, and medical history (*p* > 0.05) (Table 3).

**Table 3.** **Comparison of general information between the FSD and non-FSD Groups [$\bar{x}$ ± s, M (Q_𝐦𝐢𝐧_, Q_𝐦𝐚𝐱_), n (%)]**.

Items		FSD	non-FSD	*t/χ^2^/z*-value	*p*-value
		(n = 69)	(n = 192)		
Age (years)		43.22 ± 6.41	43.31 ± 5.31	0.12	0.91
BMI (kg/m^2^)		22.50 (18.40, 25.10)	22.10 (18.90, 25.10)	1.63	0.10
Menstrual status	Normal	46 (66.67%)	152 (79.17%)	8.76	0.01
	Amenorrhea	8 (11.59%)	24 (12.50%)		
	Disorder	15 (21.74%)	16 (8.33%)		
Educational level	Secondary school and below	41 (59.42%)	105 (54.69%)	0.46	0.50
	University and above	28 (40.58%)	87 (45.31%)		
Monthly household income	<$822	48 (69.57%)	129 (67.19%)	0.13	0.72
	≥$822	21 (30.43%)	63 (32.81%)		
Marriage	Yes	54 (78.26%)	162 (84.38%)	1.33	0.25
	No	15 (21.74%)	30 (15.63%)		
Childbirth	Yes	59 (85.51%)	125 (65.10%)	10.16	0.001
	No	10 (14.49%)	67 (34.90%)		
Medical history	Yes	48 (69.57%)	154 (80.21%)	3.29	0.07
	No	21 (30.43%)	38 (19.79%)		

### Comparison of Marital Status and Psychological State between the FSD and Non-FSD Groups

Olson Marital Quality Questionnaire analysis revealed significant differences between the FSD and non-FSD groups in marital satisfaction, compatibility, spousal communication, conflict resolution methods, and sexual life (*p* < 0.05). However, no significant differences were observed between the two groups regarding idealization, economic arrangements, leisure activities, children’s lives, relationships with friends and family, role equality, and shared beliefs (*p* > 0.05). Additionally, the FSD group showed significantly higher HAMD and HAMA scores than the non-FSD group (Table 4).

**Table 4.** **Comparison of marital status and psychological well-being between the two groups [$\bar{x}$ ± s]**.

Items	FSD	non-FSD	*t*-value	*p*-value
	(n = 69)	(n = 192)		
Idealization	39.25 ± 4.92	39.69 ± 8.95	0.39	0.70
Marital satisfaction	34.26 ± 5.85	36.14 ± 4.73	2.65	0.01
Compatibility	32.67 ± 5.49	35.20 ± 7.00	2.71	0.01
Spousal communication	32.17 ± 4.77	33.92 ± 6.54	2.03	0.04
Conflict resolution methods	31.23 ± 4.00	34.95 ± 4.22	6.36	<0.001
Economic arrangements	34.15 ± 5.60	34.69 ± 6.50	0.62	0.54
Leisure activities	34.26 ± 6.54	35.03 ± 5.60	0.93	0.35
Sexual life	33.51 ± 6.64	36.58 ± 5.75	3.65	<0.001
Children’s life	35.71 ± 5.48	35.54 ± 6.31	0.20	0.84
Relationships with friends and family	37.77 ± 5.38	37.92 ± 5.63	0.20	0.84
Role equality	30.81 ± 4.32	30.51 ± 4.41	0.49	0.63
Shared beliefs	31.06 ± 5.05	31.33 ± 5.44	0.36	0.72
Total score	408.41 ± 20.48	421.42 ± 21.76	4.33	<0.001
HAMD score	15.01 ± 3.27	13.81 ± 3.65	2.42	0.02
HAMA score	13.19 ± 2.95	11.47 ± 2.00	4.51	<0.001

Note: HAMD, Hamilton Depression Rating Scale; HAMA, Hamilton Anxiety Rating Scale.

### The Multivariate Logistic Regression Analysis of Risk Factors for FSD in Schizophrenia Women

A binary logistic regression analysis was performed to identify risk factors for FSD in women with schizophrenia during the recovery period. The results indicated that factors such as childbirth, marital satisfaction, conflict resolution, sexual life, anxiety, and depression were significant predictors of FSD (*p* < 0.05). Specifically, women who had given birth were more likely to experience FSD (Odds Ratio (OR) = 2.531, *p* = 0.044). Furthermore, lower marital satisfaction (OR = 0.886, *p* = 0.001), inadequate conflict resolution (OR = 0.816, *p* < 0.001), and dissatisfaction with sexual life (OR = 0.929, *p* = 0.016) were associated with an increased risk of FSD. Additionally, higher HAMD scores (OR = 1.116, *p* = 0.035) and HAMA scores (OR = 1.459, *p* < 0.001) demonstrated a strong association between FSD and psychological distress. Moreover, menstrual status, analyzed as a categorical variable (normal, menopausal, and irregular), indicated no significant association with FSD (Table 5).

**Table 5.** **Binary logistic regression analysis of risk factors for FSD in schizophrenic women**.

Factor	β	SE	Wald χ^2^ value	*p*-value	OR	95% CI
Constant	8.789	2.471	10.283	0.001	6564.435	
Menstruation	Ref		3.143	0.208		
Menstruation (1)	0.002	0.570	0.00	0.997	1.002	0.328–3.062
Menstruation (2)	0.885	0.507	3.046	0.081	2.424	0.897–6.551
Childbirth	0.928	0.461	4.050	0.044	2.531	1.025–6.250
Marriage satisfaction	–0.122	0.037	10.997	0.001	0.886	0.824–0.952
Personality compatible	–0.041	0.027	2.373	0.123	0.960	0.911–1.011
Spousal communication	–0.057	0.032	3.208	0.073	0.945	0.888–1.005
Conflict resolution methods	–0.203	0.048	17.868	<0.001	0.816	0.743–0.897
Sexual life	–0.073	0.030	5.804	0.016	0.929	0.876–0.986
HAMD score	0.110	0.052	4.439	0.035	1.116	1.008–1.236
HAMA score	0.378	0.087	18.974	<0.001	1.459	1.231–1.729

SE, Standard Error; OR, Odds Ratio; CI, Confidence Interval; Menstruation (1), disorder; Menstruation (2), amenorrhea.

## Discussion

Schizophrenia is a complex mental disorder characterized by severe disturbances in thoughts, emotions, and behavior. It has multifaceted impacts on patient’s physical, psychological, and social functioning. In female patients, sexual dysfunction is a common concern during the rehabilitation process [21]. Sexual dysfunction may not only result from treatment like antipsychotic medication but also significantly affect their mental health, intimate relationships, and social functioning, thereby hindering the recovery process [22]. This study aims to investigate the characteristics and contributing factors of sexual dysfunction in female schizophrenia patients during rehabilitation, providing valuable insights for clinical interventions.

The results revealed that the primary manifestations of sexual dysfunction in female schizophrenia patients during rehabilitation include reduced libido, orgasmic disorder, and decreased sexual satisfaction. Further analysis indicated psychological health, reproductive history, and marital satisfaction as significant factors affecting sexual function. Notably, the high prevalence of postpartum sexual dysfunction may be closely associated with substantial hormonal fluctuations [23]. The rapid decline in estrogen and progesterone levels after childbirth can directly reduce libido, while pain from childbirth, physical changes during postpartum recovery, and the challenges of adjusting to maternal roles may exacerbate negative self-perception and psychological stress [24]. Additionally, due to the diminished social adaptability associated with schizophrenia, patients are more vulnerable to the combined pressures of maternal responsibilities and marital roles. Studies have shown that professional psychological interventions during the postpartum period can help patients adjust their self-perceptions, alleviate postpartum anxiety and depressive symptoms, and promote sexual function recovery, thereby enhancing self-confidence and social functioning [25, 26]. Restoring sexual function can significantly improve overall psychological health, accelerate disease recovery, and help patients better adapt to their social roles, promoting long-term rehabilitation.

Anxiety and depression, common comorbid symptoms of schizophrenia, were significantly associated with sexual dysfunction [27]. Anxiety may suppress libido by worsening anticipatory anxiety regarding sexual activity, while depression can indirectly affect sexual function by reducing overall life satisfaction [28]. Further analysis revealed that negative perceptions of sexual function may reinforce anxiety and depressive states, forming a vicious cycle. For patients in the rehabilitation phase, dealing with multiple challenges such as the risk of relapse, impaired social functioning, and medication side effects, the negative impact of psychological health on sexual function is particularly pronounced [29]. Therefore, interventions targeting psychological health are crucial in improving sexual dysfunction [30]. By effectively alleviating anxiety and depression, psychological interventions can not only enhance sexual function but also improve overall quality of life, further promoting recovery.

Marital satisfaction is another crucial factor influencing sexual function [31]. This study found that the quality of marital relationships has a potential protective effect on patients’ sexual function. Mainly, when communication between spouses is effective and emotional support is sufficient, a stable marital relationship can significantly enhance patients’ psychological security, thereby improving sexual function. However, due to emotional instability and impaired social skills associated with schizophrenia, patients often face greater challenges in marital relationships [29]. Studies have highlighted the positive impact of family support on recovery [32, 33]. Providing systematic marital counseling and sexual education for patients and their spouses could improve marital relationships, elevate sexual satisfaction, and indirectly alleviate sexual dysfunction. A healthy marital relationship not only facilitates the restoration of sexual function but also strengthens the social support network, further promoting overall recovery.

While this study identified several key factors influencing sexual dysfunction in rehabilitating female schizophrenia patients, it has certain limitations. For example, it did not thoroughly analyze the specific effects of the different types and dosages of antipsychotic medications on sexual function, nor did it fully consider the impact of disease duration and socioeconomic and cultural backgrounds. Future research should incorporate these medication-related factors and sociocultural dimensions, using larger, multicenter studies to validate the findings and elucidate the underlying mechanisms. These efforts will enhance the understanding of sexual dysfunction in schizophrenia patients and provide a scientific basis for developing more comprehensive and targeted clinical intervention strategies. Additionally, research that combines multidimensional factors may uncover potential pathways of sexual dysfunction in the schizophrenia rehabilitation process, providing novel avenues for clinical practice. This will not only improve overall quality of life but also contribute to optimizing schizophrenia rehabilitation management strategies.

## Conclusion

In summary, fertility status, anxiety, depression, marital satisfaction, conflict resolution methods, and sexual life quality are independent influencing factors for sexual dysfunction in schizophrenia women in the recovery phase. Interventions addressing these factors can significantly improve sexual dysfunction, thereby promoting comprehensive recovery in these patients.

## Availability of Data and Materials

The data used and/or analyzed during the current study are available from the corresponding author.

## References

[1] Owen MJ, Sawa A, Mortensen PB (2016). Schizophrenia. *Lancet (London, England)*.

[2] Jester DJ, Thomas ML, Sturm ET, Harvey PD, Keshavan M, Davis BJ (2023). Review of Major Social Determinants of Health in Schizophrenia-Spectrum Psychotic Disorders: I. Clinical Outcomes. *Schizophrenia Bulletin*.

[3] Gu YW, Fan JW, Zhào H, Zhao SW, Liu XF, Yu R (2024). Imaging Transcriptomics of the Brain for Schizophrenia. *Alpha Psychiatry*.

[4] Stępnicki P, Kondej M, Kaczor AA (2018). Current Concepts and Treatments of Schizophrenia. *Molecules (Basel, Switzerland)*.

[5] Barker LC, Vigod SN (2020). Sexual health of women with schizophrenia: A review. *Frontiers in Neuroendocrinology*.

[6] Huang YH, Hou CL, Ng CH, Chen X, Wang QW, Huang ZH (2019). Sexual dysfunction in Chinese rural patients with schizophrenia. *BMC Psychiatry*.

[7] Zhang L, Chen Y, Sun Y, Zhou Y, Li Q, Jia Y (2024). Prevalence of sexual dysfunction in Chinese patients with schizophrenia: a systematic review and meta-analysis. *Sexual Medicine*.

[8] Kumari P, Kumar R, Rohilla J (2024). Sexual dysfunction, marital relationship, and subjective quality of life among women with schizophrenia: Analytical case-control study. *Indian Journal of Psychiatry*.

[9] Zeleke FT, Ezedin S, Aleminew F, Alem KG, Tefera DT, Demissie M (2023). Sexual dysfunction and its associated factors among reproductive-age women at Gurage Zone, Southern Ethiopia, 2023. *BMC Public Health*.

[10] Maya ET, Boamah MO, Agyabeng K, Srofenyoh E, Mumuni K, Samba A (2023). Determinants of sexual dysfunction in pregnancy in a large tertiary hospital in Ghana. *PloS One*.

[11] Khajoei Nejad F, Rafati F, Rafati S, Dastyar N (2023). The association between sexual function, quality of marital relationship and associated factors in women with a history of ectopic pregnancy: a cross-sectional study in Iran. *BMC Women’s Health*.

[12] Malaekah H, Al Medbel HS, Al Mowallad S, Al Asiri Z, Albadrani A, Abdullah H (2022). Prevalence of pelvic floor dysfunction in women in Riyadh, Kingdom of Saudi Arabia: A cross-sectional study. *Women’s Health (London, England)*.

[13] Ozdemir S, Yilmaz B, Karasahin KE, Yuksel C (2024). Coping styles for sexual dysfunction and stress among married women of reproductive age: a cross-sectional study from Turkiye. *JPMA. The Journal of the Pakistan Medical Association*.

[14] Plotogea M, Zgura A, Mehedințu C, Scurtu F, Petca A, Varlas VN (2023). Women’s Sexual Dysfunctions Following Stem Cell Transplant and the Impact on Couple Relationship. *Life (Basel, Switzerland)*.

[15] Sabanagic-Hajric S, Memic-Serdarevic A, Sulejmanpasic G, Mehmedika-Suljic E (2022). Influence of Sociodemographic and Clinical Characteristics on Sexual Function Domains of Health Related Quality of Life in Multiple Sclerosis Patients. *Materia Socio-medica*.

[16] Simon JA, Clayton AH, Goldstein I, Kingsberg SA, Shapiro M, Patel S (2022). Effects of Flibanserin on Subdomain Scores of the Female Sexual Function Index in Women with Hypoactive Sexual Desire Disorder. *Sexual Medicine*.

[17] Rosen R, Brown C, Heiman J, Leiblum S, Meston C, Shabsigh R (2000). The Female Sexual Function Index (FSFI): a multidimensional self-report instrument for the assessment of female sexual function. *Journal of Sex & Marital Therapy*.

[18] Hamilton M (1967). Development of a rating scale for primary depressive illness. *The British Journal of Social and Clinical Psychology*.

[19] HAMILTON M (1959). The assessment of anxiety states by rating. *The British Journal of Medical Psychology*.

[20] Fowers BJ, Olson DH (1989). Enrich marital inventory: a discriminant validity and cross-validation assessment. *Journal of Marital and Family Therapy*.

[21] Fares J, Mohamed F, Yosra Z, Oumaya H, Riadh B, Jihenne M (2023). Influence of Antipsychotic Agents on the Sexuality of Patients Diagnosed with Schizophrenia. *Current Therapeutic Research, Clinical and Experimental*.

[22] Barut A, Mohamud SA (2023). The psychosexual and psychosocial impacts of polygamous marriages: a cross-sectional study among Somali women. *BMC Women’s Health*.

[23] Gutzeit O, Levy G, Lowenstein L (2020). Postpartum Female Sexual Function: Risk Factors for Postpartum Sexual Dysfunction. *Sexual Medicine*.

[24] Tavares CSS, Gomes Dos Santos Oliveira SJ, de Gois-Santos VT, Vaez AC, de Menezes MO, Santos HP (2021). Quality of life, depressive symptoms, anxiety, and sexual function in mothers of neonates with congenital syphilis in the Northeast Brazil: A cohort study. *Lancet Regional Health. Americas*.

[25] Erfanifar E, Behroozi N, Latifi SM, Abbaspoor Z (2022). The effectiveness of cognitive-behavioural consultation on sexual function and sexual self-efficacy of women after childbirth. *European Journal of Obstetrics & Gynecology and Reproductive Biology: X*.

[26] Zamani M, Latifnejad Roudsari R, Moradi M, Esmaily H (2019). The effect of sexual health counseling on women’s sexual satisfaction in postpartum period: A randomized clinical trial. *International Journal of Reproductive Biomedicine*.

[27] Saleh SA, Almadani N, Mahfouz R, Nofal HA, El-Rafey DS, Seleem DA (2024). Exploring the Intersection of Depression, Anxiety, and Sexual Health in Perimenopausal Women. *International Journal of Women’s Health*.

[28] Fiala L, Lenz J, Bob P (2021). Effect of psychosocial trauma and stress on sexual dysfunction in women with endometriosis. *Medicine*.

[29] Guo Y, Qu S, Qin H (2018). Study of the relationship between self-stigma and subjective quality of life for individuals with chronic schizophrenia in the community. *General Psychiatry*.

[30] Azin SA, Golbabaei F, Warmelink JC, Eghtedari S, Haghani S, Ranjbar F (2020). Association of depression with sexual function in women with history of recurrent pregnancy Loss: descriptive-correlational study in Tehran, Iran. *Fertility Research and Practice*.

[31] Penubarthi S, Kailash SZ, Sureshkumar K, Rajalakshmi AK (2022). Sexual Dysfunction in Remitted Female Patients with Depression on SSRIs: Associated Factors and Relation to Marital Satisfaction and Quality of Life. *Indian Journal of Psychological Medicine*.

[32] Tahan M, Saleem T, Moshtagh M, Fattahi P, Rahimi R (2020). Psychoeducational Group Therapy for sexual function and marital satisfaction in Iranian couples with sexual dysfunction disorder. *Heliyon*.

[33] Guercio C, Mehta A (2018). Predictors of Patient and Partner Satisfaction Following Radical Prostatectomy. *Sexual Medicine Reviews*.

